# Inside anticancer therapy resistance and metastasis. Focus on CD36

**DOI:** 10.7150/jca.90457

**Published:** 2024-01-27

**Authors:** Ioana M. Lambrescu, Gisela F. Gaina, Laura C. Ceafalan, Mihail E. Hinescu

**Affiliations:** 1 Cell Biology, Neurosciences, and Experimental Myology Laboratory, Victor Babeș Institute of Pathology, 050096 Bucharest, Romania.; 2 Department of Cellular and Molecular Biology and Histology, Carol Davila University of Medicine and Pharmacy, 050474 Bucharest, Romania.; 3 National Institute of Pathology "Victor Babes," 050096 Bucharest, Romania.

**Keywords:** CD36, cancer treatment, drug resistance, chemotherapy, lipid metabolism

## Abstract

Despite recent advances in targeted cancer therapies, drug resistance remains an important setback in tumor control. Understanding the complex mechanisms involved in both innate and acquired drug resistance represents the first step in discovering novel therapeutic agents. Because of its importance in tumorigenesis, progression, and metastasis, lipid metabolism is increasingly garnering attention. CD36 is a membrane receptor at the top of the signaling cascade that transports lipids. Its expression has been repeatedly presented as an unfavorable prognostic factor for various tumor types, raising the question: could CD36 be a critical factor in cancer treatment resistance? In our review, we set out to explore the most prominent studies on the implication of CD36 in resistance to platinum-based drugs and other adjuvant cancer therapies in solid and haematological neoplasia. Moreover, we provide an overview of the latest anti-CD36 cancer therapies, thus opening new perspectives for future personalized medicine.

## Introduction

The importance of lipid metabolism in tumor initiation, growth, and metastasis is receiving much attention in cancer research. To fulfill the demands for rapid proliferation and development, tumor cells can potentially increase lipid accumulation and metabolism 1. CD36 is a transmembrane glycoprotein of the class B scavenger receptor family, also known as Fatty Acid translocase (FAT), platelet GPIV, or GP88, that interacts with a variety of ligands such as apoptotic cells, thrombospondin-1 (TSP-1) and fatty acids (FAs) 2,3.

**Figure 1:**
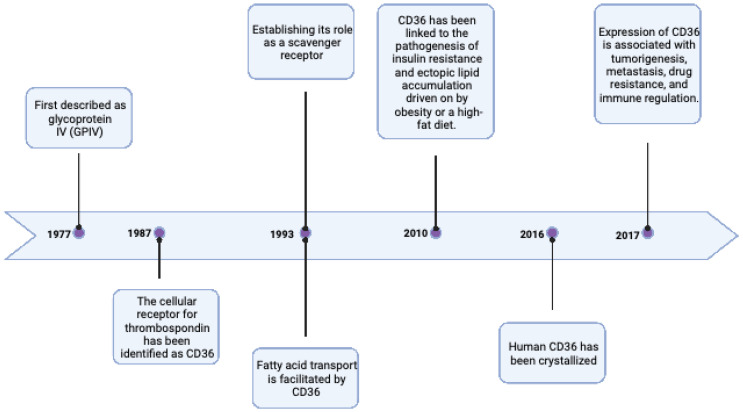
The most significant historical milestones of CD36. Created with BioRender.com

The history of CD36 goes back to 1977 when it was first described by Clemstone and colleagues 4. The past four decades have been extremely generous in terms of scientific research into the influence of CD36 on FA transport, insulin resistance, and tumorigenesis, as stated in Figure 1. Created with BioRender.com.

Due to the extensive glycosylation, the 472 amino acid protein has an apparent molecular mass of 88 kDa with two membrane-spanning domains and many palmitoylation sites 5. Numerous non-immune cells, such as platelets, immature erythrocytes, adipocytes, myocytes, some specialized epithelial cells, and microvascular endothelial cells, as well as a wide range of innate and adaptive immune cells, including macrophages, monocytes, dendritic cells, and subsets of T and B cells, express CD36 on their surfaces 6.

According to a thorough evaluation of several omics data from The Cancer Genome Atlas (TCGA), fatty acid (FA) metabolism is one of the most commonly altered pathways in lipid metabolism in pan-cancer 7. Furthermore, tumor immunological tolerance and carcinogenesis can be linked to CD36-driven lipid metabolic reprogramming and the function of tumor-associated immune cells 8.

Drug resistance is a significant drawback for cancer patients regarding disease control. Tumor heterogeneity and the diversity of their milieu could explain therapy resistance. Furthermore, 90% of chemotherapy failures occur during the invasion and metastatic spread of the malignancy due to drug resistance 9. Several mechanisms were suggested through which cancer cells are either innately resistant to medication treatments or develop resistance to drug therapies after exposure. Molecular alterations in drug efflux pumps, drug-metabolizing enzymes, and apoptotic regulating pathways have been found to protect tumor cells against chemotherapy 10.

Consequently, these processes may influence the drug molecule itself or its target 11. Many solid tumors, including ovarian, gastric, and oral cancers, have CD36 levels that are significantly upregulated 3,12,13. However, tumoral stroma and microvessels show downregulation in CD36 expression compared to the surrounding tissues 1, as demonstrated for breast cancer 14.

**Figure 2:**
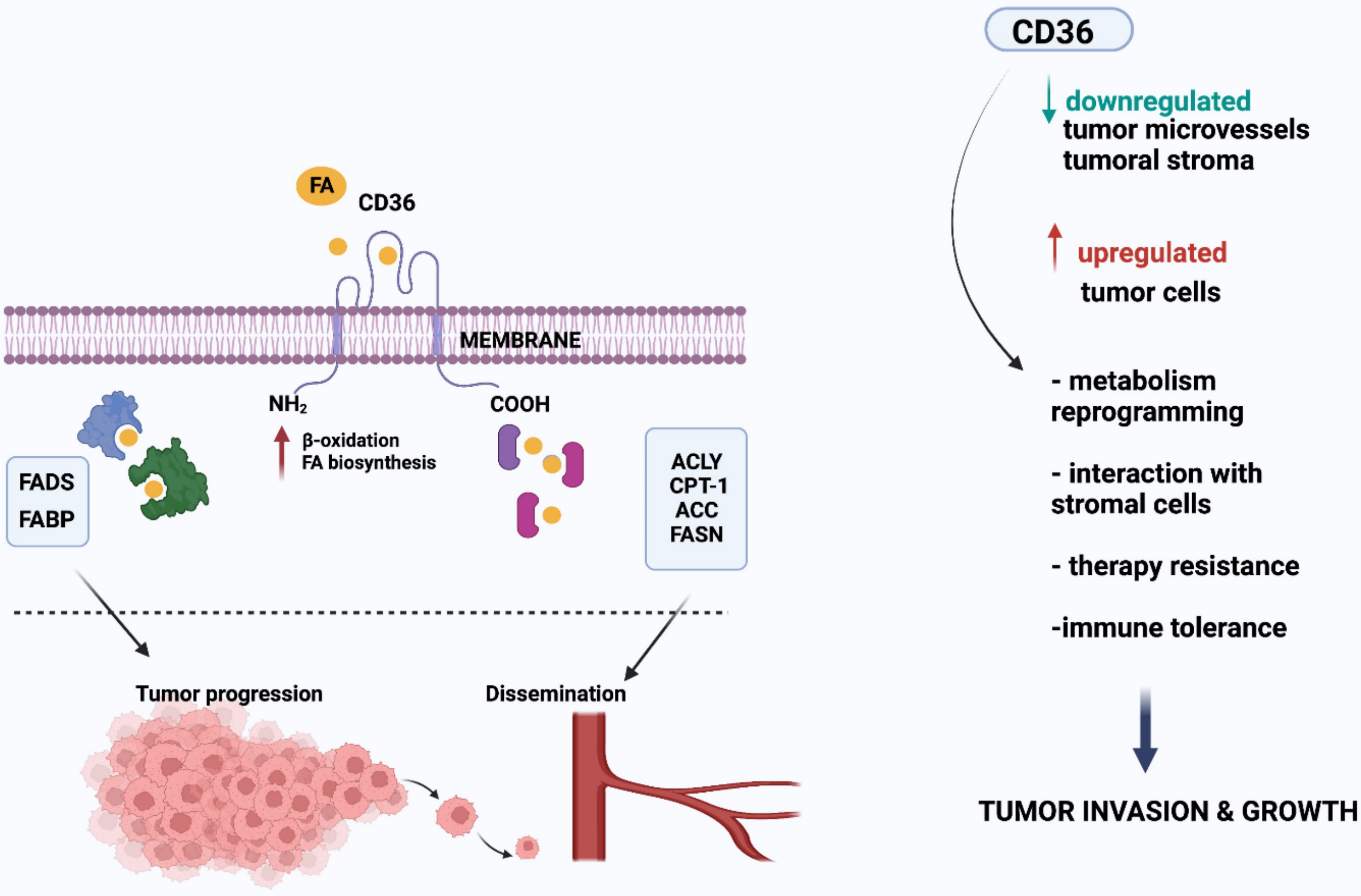
CD36 contributes to the growth and spread of tumors by boosting lipid absorption and fatty acid (FA) oxidation. Several proteins, notably fatty acid binding protein (FABP) and fatty acyl-CoA desaturases (FADS), coordinate tumor cell development. As seen on the right side of the picture, lipid metabolism alterations may also affect cell motility, which may ultimately lead to metastasis. Several enzymes, including ATP citrate lyase (ACLY), carnitine palmitoyl transferase 1 (CTP-1), acetyl-CoA carboxylase (ACC), and fatty acid synthase (FASN), are involved in this process. In addition, FA are synthesized from scratch, and FASN is overexpressed in a range of cancer types. Created with BioRender.com

CD36 expression has been repeatedly presented as an unfavorable prognostic factor for various tumor types, such as ovarian and colon cancer 3,15,16. Recently, further studies have linked CD36 to tumor progression and treatment resistance via increased lipid uptake and FA oxidation (FAO) for ATP production 1,17-20 (Figure 2).

In 2017, Dong and colleagues published an exciting study that linked the progression of estrogen receptor (ER) positive breast cancer in female mice to diet-induced obesity (DIO) and the lysophosphatidic acid/protein kinase D1 (LPA/PKD-1)-CD36 signaling axis 21. According to this study, tumor angiogenesis may be driven by the downregulation of CD36 in the tumor endothelium. This is critical because targeting this pathway might represent a future therapeutic approach 21.

This review aims to highlight the impact of CD36 on cancer therapies with an emphasis on drug resistance and the direct targeting of CD36 for improving cancer control.

## The crosstalk between CD36, metastasis and drug resistance

The study of tumor biology has shown that malignancies are not produced by a single cluster of cells but rather by various cellular populations 22. The diversity of cells in the tumor microenvironment (TME) and the genetic variability could help explain this tumor heterogeneity. A tumor comprised of distinct cell populations in terms of behavior and response to treatment defines the concept of intratumoral heterogeneity 23. Furthermore, various cellular subpopulations are responsible for building the groundwork for inter- and intrametastatic heterogeneity. This is a significant deterrent to therapy and a potentiator of acquired resistance 22,24.

Cancer cells that have been exposed to chemotherapy or radiation over an extended period may become resistant to therapy, resulting in recurrence and a poor prognosis. Resistance to cancer drugs can be intrinsic. However, acquired resistance in tumor cells is more common 25.

Increased expression of proteins involved in the epithelial-mesenchymal transition (EMT) has been linked to drug resistance, thus enhancing cell migration and metastasis. In 2015, Deng and colleagues reported an interplay between CD36 and TGF-β to drive EMT in cervical cancer cells 26. Additionally, breast cancer cells acquire FAs from adipocytes via CD36 trafficking, inducing EMT 27. Finally, CD36 was shown to promote EMT through the PI3K/AKT/mTOR pathway in gastric cancer with peritoneal metastasis (PM) 28.

### CD36 and metastatic potential

CD36 seems to be more involved in the metastatic process rather than promoting the development of the primary tumor 29. Furthermore, the CD36 gene was discovered to be often amplified in metastatic cancers, with the worst survival rates in the high-copy number group 15.

The metastatic potential of CD36 was also evaluated by Pheiler and colleagues in 2018. The authors reported that CD36 is a key modulator of immune cell engulfment of tumor microvesicles, stressing the role of CD36 in transporting tumor microvesicles to the perivascular region, resulting in premetastatic cell clusters 30.

FAT/CD36 is upregulated and increases FA uptake under tumor-derived cytokines signaling in infiltrating polymorphonuclear myeloid-derived suppressor cells (MDSC). MDSC block anti-tumor T-cell responses, thus promoting tumor growth 31. The genetic depletion of CD36 limits oxidative metabolism and the induction of the immunosuppressive mechanisms, resulting in a CD8^+^ T cell-dependent delay in tumor development 31. These findings highlight the importance of CD36 in immune suppression and tumor development, which further recommends CD36 as a potential therapeutic target, especially for metastatic cancers.

Metastasis-initiating cells respond to dietary lipids. High-fat diets or high quantities of palmitic acid (PA) significantly increase the metastatic potential. *In vivo*, studies on both immunocompetent and immunodeficient mouse strains revealed that PA or a high-fat diet enhances the ability of CD36^+^ metastasis-initiating cells to proliferate 32. Moreover, Pascual et al. proved that the number and size of metastases were reduced by CD36 inhibition or knockdown, as well as by employing cancer cells expressing mutant CD36 32.

Finding novel treatment strategies for patients with metastases is now of utmost importance. The liver is among the most typical sites for metastatic illness, and patients who progress to this stage have a poor prognosis and reduced therapy response. Although immunotherapy for cancer is an influential and rapidly expanding therapeutic option, approximately 15% to 20% of patients with liver metastases benefit from it 33,34.

One of the reasons immunotherapy is ineffective in metastatic liver disease is the accumulation of macrophages at this level. Recent evidence suggests that one of the factors promoting tumor growth could be CD36-mediated lipid metabolism of the tumor-associated immune cells' 35.

Thus, one potential therapeutic strategy could focus on CD36 inhibition to target the tumor-macrophage metabolic interface. In this regard, it was established in preclinical animal models that blocking CD36 in macrophages restores CD8^+^ T cell immunity and reduces liver metastases 34.

## Dysregulation of lipid metabolism and anticancer drug resistance

It has been hypothesized that the metabolic switch that enables cancer cells to adapt to treatment-induced cellular stress could be the abnormal lipid metabolism. Several intricate mechanisms are responsible for the dysregulation of lipid metabolism in cancer. De novo lipid synthesis or lipogenesis, FAO in cancer cell mitochondria, and the coexistence of lipolysis and lipogenesis in cancer cells come together to meet the needs of tumor cell proliferation and growth 36. FAs are coupled to fatty acid binding proteins (FABPs) in the cytoplasm and subsequently metabolized to acyl-CoA. Carnitine palmitoyltransferase 1 (CPT1) is required to transport acyl-CoA into the mitochondria and represents the major rate-limiting enzyme for FAO 36,37.

Numerous posttranslational modification sites on CD36 have been identified. These modifications, which control CD36 stability, protein folding, and translocation, can be glycosylated, phosphorylated, palmitoylated, acetylated, or ubiquitylated. This causes CD36 to become involved in several signaling pathways once ligands bind at various subcellular sites 1. These mechanisms are particularly essential since they serve as the basis for developing novel therapeutic lines that may be used to combat resistance to already approved drugs.

The increased FA uptake by breast cancer cells due to the up-regulation of CD36 expression can be demonstrated by co-culturing these cells with adipocytes 27. Thus, the uptake of FA released by adipocytes provides the energetic requirements for cancer cell survival 38-42. Moreover, the adipocyte-derived interleukin-6 (IL-6) and leptin regulate epithelial-mesenchymal transition (EMT) in cancer cells and supports stem cell renewal and chemoresistance 43-47.

Anticancer therapy may lead to an enrichment of a pre-existing cellular subpopulation exhibiting an abnormal lipid metabolism, which is thought to represent the cancer stem cells (CSCs). It has been observed that depending on cancer cell type, CSCs can undergo various changes in their lipid metabolism, such as an increase in de novo lipogenesis or lipid uptake 36. According to Yasumoto et al., de novo lipogenesis appears more active in glioma stem cells (GSCs) than in differentiated bulk cells. The authors discovered that GSCs have higher levels of ^14^[C]-glucose and ^14^[C]-acetate incorporation into lipids in comparison with non-GSCs 48. Additionally, in various cancer cell types, including glioma, pancreatic tumors, and breast cancer, fatty acid synthase (FASN) was identified as an essential factor in CSC survival 49.

A substantial increase in lipid droplet concentration and stearoyl-CoA desaturase-1 expression was seen in non-small cell lung cancer (NSCLC) tumor samples from patients before and after Gefitinib therapy 50. Furthermore, cytarabine (AraC)-treated acute myeloid leukemia cells have altered lipid metabolism, as demonstrated by an increased CD36 expression and mitochondrial FAO 51. Thus, another critical point in maintaining the CSC pool could be represented by FAO. Two significant publications highlighted this concept focusing on the pharmacological inhibition of FAO by etomoxir (irreversible inhibitor of CTP-1) that either decreased the number of leukemic stem and progenitor cells or sensitized the CSCs to sorafenib, a tyrosine kinase inhibitor 52,53.

Another mechanism through which lipid metabolism can be influenced is via HIF-2α. This member of the hypoxia-inducible factor family is responsible for the proliferation, angiogenesis, and pharmacologic resistance in various types of cancer 54. One of the proposed mechanisms suggests that the increased expression of HIF-2α in tumor cells may increase the expression of PLIN2 - a cytosolic lipid droplet coat protein frequently used as a marker of intracellular lipid accumulation 55,56. Overexpression of PLIN2 is sufficient to enhance lipid production and storage in murine fibroblast *in vitro* and in the liver *in vivo* 55,57. Interestingly, in mouse hepatocytes, *in vivo*, PLIN2 expression is associated with HIF-2 activation.

Moreover, in another study, microarray data indicated that HIF-2 enhances PLIN2 mRNA expression *in vitro* in clear cell renal carcinoma 58,59. Thus, PLIN2 expression driven by HIF-2α can promote tumorigenesis and pharmacologic resistance to endoplasmic reticulum stress 58. Consequently, it could be interesting to investigate further if CD36 expression is influenced by hypoxia in the tumor environment.

## The modulation of CD36 in various cancer therapies with emphasis on drug resistance

Changes in lipid metabolism have long been linked to resistance to traditional chemotherapies and targeted treatments for various malignancies. Thus, in the context of personalized medicine, many studies have focused over the last two decades on CD36, as it is one of the most reputable FA transporters.

Most studies suggest that the lipid metabolism of cancer-resistant cells varies in response to therapy and environmental and cellular contexts 36. In the following section, we present the most prominent studies of the past decade focusing on the modulation of CD36 in different cancer settings emphasizing drug resistance (Table 1).

### Breast cancer

Adipocytes influence breast cancer behavior, as these cells release FAs into the tumor microenvironment. When human adipocytes and breast cancer cells were co-cultured, the expression of CD36 was increased 27. Furthermore, the hormone leptin, released by adipocytes, promotes cancer stem cell renewal and chemoresistance 46,47.

In 2007, the group conducted by Vasquez-Martin showed that due to the bidirectional crosstalk between the FASN and the human epidermal growth factor receptor-2 (HER2), cancer cells undergo apoptosis as the endogenous FA lipogenic pathway is inhibited 68. According to this study, FASN-driven cellular signaling is implicated in HER2-induced malignant transformation, and FASN blockage suppresses HER2 transcription 68.

More than ten years later, Feng and colleagues discovered that breast cancer cells could escape lapatinib (an oral dual tyrosine kinase inhibitor that targets epidermal growth factor receptor-EGFR and HER2) and consequently survive by displaying a change toward CD36-mediated FA uptake 61,69. This study aimed to determine whether lapatinib-resistant cells developed resistance over time or were formed from an innately resistant subpopulation expressing high levels of CD36. The authors concluded that after continuous lapatinib therapy, drug-naïve cells significantly increased CD36 expression rather than a selection process of resistant cells, which already exhibited a CD36 overexpression 61.

Although Tamoxifen is a standard treatment used in patients with ER-positive breast cancer, oncologists must be aware that therapy resistance can develop in some instances. Liang and colleagues published an interesting study demonstrating that Tamoxifen's influence on the proliferation of ER-positive MCF-7 cells was connected to regulating CD36 expression. Additionally, the authors showed that Tamoxifen's growth inhibitory impact was decreased by activating CD36 expression, whereas siRNA-mediated CD36 inhibition caused a synergistic suppression of cell growth 62.

Due to its poor prognosis and aggressive nature, triple-negative breast cancer (TNBC), which lacks estrogen, progesterone, and human epidermal growth factor receptor 2, represents a therapeutic challenge 70. Thus, finding appropriate therapy combinations with the lowest risk of toxicity is critical in this scenario.

Genistein is a classic example of a phytoestrogen molecule, similar to other plant components like lignans, with estrogenic action 71. Furthermore, this compound is crucial for the phospholipid breakdown of MDA-MB-231 cells (TNBC cell line that is highly aggressive, invasive, and poorly differentiated) and tyrosine kinase signal transduction pathways 63. A crucial metabolic mechanism for the development of TNBC is FA oxidation. Consequently, CD36 targeting may be a possible TNBC treatment approach. Exploring this pathway, using nanomaterials such as siRNA carriers in combination with Genistein in MDA-MB-231 cells revealed a significant reduction of CD36 expression through phosphorylation of the p38 MAPK pathway, resulting in cell growth inhibition 63. Nobilet is another flavonoid whose effectiveness has been tested in the context of ER-positive breast cancer cells. This compound reduced tumor angiogenesis, modulating Src, FAK, and STAT3 signaling through PXN 72. In 2017, Sp and colleagues hypothesized that Nobiletin's antiangiogenic effect included regulation of CD36 through signal transducer and activator of transcription 3 (STAT3) rather than via TSP-1 since CD36 also interacts with the oncogene STAT3. The authors demonstrated that CD36 siRNA decreased cell invasion by over 65% in the MDA-MB-231 cell line, indicating that CD36 plays a role in tumor metastasis 64.

### Pulmonary cancer

Lung cancer is the most common cause of cancer death worldwide; Non-Small Cell Lung Cancer (NSCLC) accounts for the majority of cases 73,74. Lipid metabolic reprogramming is one of the leading mechanisms for cancer development, progression, and resistance to therapies 73. The targeting of HMG-box transcription factor 1 (HBP1) by miR-21 has previously been demonstrated to promote the invasion and migration of drug-resistant lung adenocarcinoma cancer cells 75. In 2019, Ni and colleagues published a study that examines the relationship between miR-21 and CD36-regulated FA metabolism in NSCLC cell lines 76. The authors demonstrated that miRNA-21 mimic treatment stimulated CD36 expression and cell proliferation and migration. Moreover, CD36 silencing decreased intracellular lipid content and hindered the development of tumor cells mediated by miRNA-21^76^.

Using weighted gene co-expression network analysis (WGCNA), Sun and colleagues identified CD36 as a hub gene with a low expression in lung cancer 65. However, they outline a new link between increased CD36 gene methylation and tumor development. The authors concluded a decrease in CD36 methylation after administering Decitabine, a DNA methylation inhibitor, and Chidamide, a particular class I histone deacetylase inhibitor, alone and in combination, ultimately inhibited tumor growth 65.

### Haematological malignancies

Many hypotheses have been proposed to elucidate chemoresistance in acute myeloid leukemia (AML). However, this phenomenon continues to represent an issue regarding survival rates. An interesting study from 2019 analyzed AraC resistance in AML patient-derived xenografts and targeted mitochondrial metabolism via the CD36-mitochondrial FA β-oxidation and oxidative phosphorylation 51. The authors identified the CD36 receptor as one of the most differentially expressed genes associated with FA and lipid metabolism in chemoresistant leukemic cells. This may open up new treatment options for AML patients with chemoresistance as inhibiting oxidative phosphorylation via CD36, might boost AraC's antileukemic impact 51.

Interestingly, the gonadal adipose tissue (GAT) can be used as a niche by a subpopulation of leukemic stem cells. This interaction increases FA metabolism, as observed in a murine model of blast crisis chronic myeloid leukemia (CML). A high level of FAO and a drug-resistant phenotype was demonstrated in CD36^+^ cells together with the capacity of GAT to protect the cells against chemotherapy 77.

Reducing drug resistance and simultaneously reducing tumor growth and dissemination can sometimes be obtained by switching from one cancer drug to another. According to this principle, Landberg and colleagues observed in CML cells that the second-generation tyrosine kinase inhibitor (TKI) nilotinib can overcome the decreased *in vitro* sensitivity of CD36-expressing cells to imatinib 78.

### Pancreatic cancer

With a 5-year survival rate of barely 6%, pancreatic ductal adenocarcinoma (PDAC) is the fourth most significant cause of cancer-related deaths globally 79. Jia and colleagues showed that in pancreatic cancer cell lines and tumor tissue, CD36 expression is considerably reduced 80. Moreover, lower TNM staging and CA19-9 levels were predicted by a low expression of CD36. However, the latter also predicted larger tumors and poor survival. This might be because CD36-negative tissue had fewer lymph node metastases and infiltrated surrounding tissue, decreasing TNM staging 80.

In gemcitabine-treated patients, CD36 expression in resected pancreatic ductal adenocarcinoma (PDAC) specimens was also correlated to cancer prognosis 20. The group led by Kubo revealed that, consequently, to CD36 down-regulation after siRNA transduction, PDAC cell resistance to gemcitabine was reduced, and anti-apoptosis proteins were inhibited, supporting the idea that chemoresistance can be fought with anti-CD36 treatments 20. The heterogeneity in patient samples may cause inconsistency between these studies regarding CD36 expression. A larger and more homogeneous cohort of samples would benefit the continuing study of CD36 expression in connection to pancreatic cancer with an emphasis on treatment resistance.

### Ovarian cancer

There is no proof yet of the involvement of CD36 in ovarian cancer development and progression. However, some studies used CD36 as a therapeutic escape for Cisplatin-resistant ovarian cancer. Resistance to platinum-based therapies and toxicity are two crucial issues that must be addressed to obtain remission without affecting the quality of life. In this regard, Pt(IV) pro-drugs that resemble the fatty acid structure (FALPs) have been developed to upregulate CD36 receptors, allowing them to enter the ovarian cancer cell. The difference between FAs (necessary for lipid metabolism) and Pt(IV) pro-drugs consists in the fact that the latter can cause mitochondrial damage, rendering them effective in fighting cisplatin resistance in ovarian cancer 67. Using a platinum-resistant ovarian cancer PDX model, a prosaposin-derived therapeutic cyclic peptide was expected to promote cancer regression 81. The authors of this study demonstrated that the prosaposin-based therapeutic agent inhibits the progression of ovarian cancer through TSP-1 and downstream CD36 signaling, which further highlights the importance of CD36 in the tumor microenvironment 81.

### Melanoma

The use of targeted treatments and immunotherapies for metastatic melanoma has evolved in the past decade, yet the vast majority of patients are still left uncured. It remains a major clinical issue to overcome resistance to treatment. A TCGA melanoma cohort research reveals that melanoma patients with high CD36 expression have a worse clinical prognosis 82. A possible explanation could be the up-regulation of CD36 in response to mitogen-activated protein kinase inhibitors (MAPKi) in BRAF-mutated melanomas 83. Both *in vitro* and *in vivo* testing demonstrated that during both adaptation and drug tolerance states, MAPKi promotes and sustains the expression of CD36 in BRAF-mutated melanomas 83. Martini and colleagues identified CD36 as a regulator of vascular mimicry (VM) in melanoma cells. The authors concluded that, as part of the tumor microenvironment, CD36 works in tandem with adhesion molecules like integrin-3 and different microenvironment components such as laminin for organizing VM channels 82.

### Glioblastoma

Glioblastoma (GBM) is the most prevalent high-grade primary malignant brain tumor with a meager survival rate. Consequently, novel therapeutic strategies are highly needed due to the poor prognosis of individuals receiving currently authorized GBM treatments 84. Highly vascularized tumors like GBMs depend on developing new tumor-associated blood vessels 85. Thus, in 2015, Choi and colleagues investigated *in vitro* and *in vivo* the antiangiogenic potential of three type-1 repeat (3TSR) domain of TSP-1 on GBM cells 66,86,87. The authors demonstrated that 3TSR sensitized GBM lines to caspase-3/7-mediated apoptosis by upregulating tumor-necrosis factor-related apoptosis-inducing ligand (TRAIL) receptor DR4/5 expression in a CD36-dependent manner. Furthermore, the results showed a decrease in tumor growth, with prolonged survival of mice bearing TRAIL-resistant GBM using the combination of engineered human mesenchymal stem cells (MSC) with 3TSR/TRAIL 66.

A crucial method by which cells identify, phagocytose, and eliminate what is unnecessary is via scavenger receptors 88. Multiple cell types in the brain, including microglia, endothelial cells, astrocytes, and neurons, use CD36 as a the scavenger receptor 89. It was hypothesized that CSCs might recognize and react to an unfavorable environment by expressing scavenger receptors as an adaptive strategy 90. In 2015, the group conducted by Hale published an exciting paper that reports the crosstalk between patient-derived and *in vivo* xenograft models of CSCs and CD36 90. The authors demonstrated the presence of oxidized phospholipids in GBM cells, which are CD36 ligands. Additionally, exposure to oxidized low-density lipoprotein increased the proliferation of CSCs, rendering this molecular mechanism an important target for future therapeutic approaches 90.

## Different settings of anti-CD36 cancer therapies - future perspectives

In terms of FA uptake, normal cells preferentially choose the exogenous sources. In cancer cells, it was established that more than 90% of FA are de novo synthesized and FASN is overexpressed in a variety of cancer types 61. Cancer cells usually display a "lipogenic phenotype" defined by both FASN overexpression and high FA biogenesis, even in the presence of circulating exogenous FA. However, CD36 is closely connected to lipid homeostasis, angiogenesis, and tumor dissemination. Therefore it was often regarded as a potential cancer therapeutic target 1. To support this idea, addressing CD36 lipid transport activity is important as the development of resistance to HER2 inhibitors is influenced by CD36 up-regulation 61. *In vitro* studies on lapatinib-resistant breast cancer cells (rBT474) revealed that the small-molecule inhibitor sulfosuccinimidyl oleate (SSO) provides pharmacological inhibition of CD36, re-sensitizing rBT474 cells to lapatinib 61. Other studies that target CD36 in the context of breast cancer 63,64 are summarised in Table 1.

In mice, the administration of neutralizing monoclonal antibodies (JC63.1) to block CD36 has been shown to inhibit gastric cancer metastases 91. An impediment to anti-tumor immunity and cancer treatment could be explained by a large number of regulatory T cells (T_reg_ cells), which is why depleting them is a promising cancer treatment. However, it must be taken into account that this therapeutic strategy is hampered by autoimmunity 92. In 2020, the group led by Wang explored the regulation of CD36 in intratumoral T_reg_ cells. YUMMI.7 melanoma-engrafted mice were treated with an anti-CD36 monoclonal antibody, which resulted in a decrease in tumor growth with a reduction of intratumoral T_reg_ cells, whereas T_reg_ cells in the spleen and draining lymph nodes remained stable 92.

The role of natural bioactive compounds in the treatment and prevention of different types of cancer has received considerable attention in the literature 59,60. Among them is the flavonoid quercetin, which is thought to be an antioxidant that scavenges free radicals 93. It has been shown that quercetin activates the miR-1254/CD36 signaling pathway, which inhibits oral squamous cell carcinoma cells' ability to survive and invade 59.

Hypercholesterolemia is a common metabolic disease that has been linked to the development of malignancies that are steroid-targeted. However, in 2021, Yang and colleagues published a study that investigates the link between hypercholesterolemia and the aggressiveness of bladder cancer, a non-steroid-cancer 94. The authors concluded that the CD36/STAT3 signaling pathway increases cancer progression on the background of hypercholesterolemia-induced oxidized LDL (ox-LDL). Considering these mechanisms, targeting the CD36/STAT3 axis could represent a future therapeutic promise for patients who associate hypercholesterolemia and urinary bladder cancer 94.

In high-risk localized disease patient-derived xenografts (PDXs), the CD36 monoclonal antibody inhibited prostate cancer development. Thus, targeting FA absorption by blocking CD36 might also be a potential treatment strategy for patients diagnosed with prostate cancer 95. Another recent study on human prostate cancer cell lines analyzed the potency of C75, a radiosensitizing FASN inhibitor 96. The authors reported an increased C75 sensitivity in cells by CD36 neutralizing antibody, which suggests that the availability of FAs enhances the effect of the radiosensitizer 96.

A founding member of the MADS-transcriptional regulator factor that may influence the pathways of certain malignant tumors is the myocyte enhancer factor 2C-genecards (MEF2C) 97. The MEF2C-CD36 pathway is mentioned in a recent study published in 2023 as a possible approach to comprehending the mechanism of tumor regulation in colorectal cancer. Thus, MEF2C inhibits tumor growth through modulation of CD36 transcription, rendering this pathway a promising therapeutic target 97.

CD36 antibody targeting was also explored as a potential therapy in the CML setting. Thus, the group conducted by Landberg demonstrated that directing human NK cells to eliminate CML cells specifically can be obtained by CD36 targeting antibodies considering the antibody-dependent cellular toxicity 78.

The future perspective of personalized medicine resides in perfecting new therapeutic strategies that combine the strengths of peptides and monoclonal antibodies. Anticancer clinical trials exploring modified peptides with a TSP-1 mimetic activity targeting CD36, such as ABT-510 in haematological and solid neoplasia, were terminated due to severe side effects and ineffective performance 98. A problem with peptides is the rapid degradation and, therefore, the need for frequent compound administration 99. To overcome this problem and extend the half-life of the TSP-1 peptide, an antibody scaffold was added, resulting in a TSR peptide mimetic-antibody fusion molecule with favorable pharmacokinetics (CVX-045, CVX-22). CVX-045 was administered in advanced solid tumors, causing a decrease in tumor microvasculature, while CVX-22 was administered in melanoma. Even though both therapies had encouraging preclinical results, CVX-045 failed a phase 1 clinical trial due to severe adverse effects 100.

## Conclusions

Despite current therapeutic advances in treating cancer patients, drug resistance continues to be one of the limiting factors. Consequently, novel treatment approaches have been created based on a better knowledge of the molecular mechanisms disrupted during the transition of a normal cell into a malignant one. CD36 is a critical participant in cancer development and progression via lipid uptake, immunological recognition, apoptosis, and anti-angiogenesis. Additionally, its prognostic value has been extensively discussed in the literature, making this FA-transporter a potential target for cancer patients. Numerous inhibitors targeting different aspects of lipid metabolism, including CD36, have been discovered and have already proved potential effectiveness.

In this review, we have highlighted the most relevant research of the past decade investigating the role of CD36 in treatment resistance in various neoplasms, either solid or haematological. Understanding the signaling pathways, cell dependencies, and the variety of molecules involved in tumor growth and metastasis opens new avenues for cancer patients once drug resistance occurs. Nevertheless, aiming at a particular molecule or mechanism involved in lipid metabolism may not be enough to induce long-term cancer cell growth inhibition and, thus, disease control.

## Figures and Tables

**Table 1:** The most significant research of the last decade focuses on regulating CD36 in various cancer contexts, with a particular emphasis on treatment resistance.

Compound	Target /Mechanism	Type of cancer	Result	Source	Ref
**Quercetin**	miR-1254/CD36 signaling pathway	Oral squamous cell carcinoma	Anti-tumor effects on proliferation and invasion	CAL-27 cell line	(59)
**Quercetin**	Regulate TSP1 by increasing the expression of CD36	Pancreatic	• Enhancing cell adhesion • Stimulating the immune response • May reduce the death rate	Drug Bank String and TCGA databases	(60)
**Gemcitabine** **siRNA**	Downregulation of CD36	Pancreatic	siCD36 suppressed Bcl-2 in Gemcitabine-resistant (GR) PDAC cell lines	Resected specimen from pancreatic ductal adenocarcinoma (GR) PDAC cell lines	(20)
**Lapatinib**	CD36-mediated metabolic rewiring	Breast	• CD36 has a key role in HER2-targeted treatment resistance • Re-sensitizing rBT474 cells to lapatinib with SSO	Experimental model: MMTV-neu, MMTV-Cre, NSG mice Human breast cancer cells: BT474, SKBR3, HCC202	(61)
**Tamoxifen** **siRNA**	Regulation of CD36 expression	Breast	• Inhibition of CD36 expression • Restoring Tamoxifen′s capacity to inhibit cell growth	ERα- positive MCF-7 cells	(62)
**Nanoparticle load-CD36 siRNA** **Genistein**	CD36/phospho-p38 MAPK axis	Breast	• Silencing the expression of CD36 • Suppressing the proliferation of MDA-MB-231 cells • Cellular apoptosis	MDA-MB-231 cell line	(63)
**Nobiletin**	CD36/ (STAT3)/NF-κB signaling axis	Breast	• Suppressing CD36 expression • Inhibiting CD36-dependent breast cancer cell migration • Inhibiting angiogenesis • Decreasing the expression of TSP-1 and TGF-β1	MCF-7, and MDA-MB-231 cell lines	(64)
**Decitabine Chidamide**	Methylation of CD36	Lung	De-methylation and re-expression of silenced CD36	Lung cancer tissue Cell line: A549, NCI-H520, Calu-1	(65)
**Tumor necrosis factor-related apoptosis-inducing ligand (TRAIL)**	Upregulation of TRAIL receptors through engagement of CD36 by three type-1 repeat (3TSR) domain of TSP1	Glioblastoma (GBM)	Inhibits tumor growth Prolonged survival of mice bearing intracranial TRAIL-resistant GBM	GBM tumor cells and GBM-associated endothelial cells, mouse models of TRAIL-resistant GBMs	(66)
**Cisplatin** **Pt(IV) pro-drugs**	Exploiting the upregulation of CD36	Ovarian	Mitochondria-damaging FA-like drugs	Cisplatin-sensitive ovarian cancer (A2780), Cisplatin-resistant (A2780cis) and (HEK293) non-cancerous cell lines	(67)
